# Supervised versus unsupervised approaches to classification of accelerometry data

**DOI:** 10.1002/ece3.10035

**Published:** 2023-05-17

**Authors:** Maitreyi Sur, Jonathan C. Hall, Joseph Brandt, Molly Astell, Sharon A. Poessel, Todd E. Katzner

**Affiliations:** ^1^ Conservation Science Global, Inc. West Cape May New Jersey USA; ^2^ Department of Biology Eastern Michigan University Ypsilanti Michigan USA; ^3^ U.S. Fish and Wildlife Service, Hopper Mountain National Wildlife Refuge Complex Ventura California USA; ^4^ Department of Biology Boise State University Boise Idaho USA; ^5^ U.S. Geological Survey, Forest and Rangeland Ecosystem Science Center Boise Idaho USA; ^6^ Present address: Radboud Institute for Biological and Environmental Sciences (RIBES) Radboud University Nijmegen The Netherlands

**Keywords:** accelerometer, animal behavior, California condor, classification, machine learning

## Abstract

Sophisticated animal‐borne sensor systems are increasingly providing novel insight into how animals behave and move. Despite their widespread use in ecology, the diversity and expanding quality and quantity of data they produce have created a need for robust analytical methods for biological interpretation. Machine learning tools are often used to meet this need. However, their relative effectiveness is not well known and, in the case of unsupervised tools, given that they do not use validation data, their accuracy can be difficult to assess. We evaluated the effectiveness of supervised (*n* = 6), semi‐supervised (*n* = 1), and unsupervised (*n* = 2) approaches to analyzing accelerometry data collected from critically endangered California condors (*Gymnogyps californianus*). Unsupervised K‐means and EM (expectation–maximization) clustering approaches performed poorly, with adequate classification accuracies of <0.8 but very low values for kappa statistics (range: −0.02 to 0.06). The semi‐supervised nearest mean classifier was moderately effective at classification, with an overall classification accuracy of 0.61 but effective classification only of two of the four behavioral classes. Supervised random forest (RF) and k‐nearest neighbor (kNN) machine learning models were most effective at classification across all behavior types, with overall accuracies >0.81. Kappa statistics were also highest for RF and kNN, in most cases substantially greater than for other modeling approaches. Unsupervised modeling, which is commonly used for the classification of a priori‐defined behaviors in telemetry data, can provide useful information but likely is instead better suited to post hoc definition of generalized behavioral states. This work also shows the potential for substantial variation in classification accuracy among different machine learning approaches and among different metrics of accuracy. As such, when analyzing biotelemetry data, best practices appear to call for the evaluation of several machine learning techniques and several measures of accuracy for each dataset under consideration.

## Graphical abstract

Despite the widespread use of advanced bio‐logging tools, the quantity of data they produce has created a need for robust analytical methods for biological interpretation. Here we evaluate the effectiveness of 6 supervised, 1 semi‐supervised, and 2 unsupervised machine learning tools for the analysis of acceleration data from critically endangered California condors. Unsupervised classification, which is commonly used for the classification of a priori‐defined behaviors in telemetry data, likely is instead better suited to the post hoc definition of generalized behavioral states.

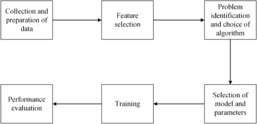

## 1 INTRODUCTION

Understanding how animals behave and move is important to improve wildlife monitoring and management, especially for species that face increasing risks in rapidly changing landscapes (Kays et al., 2015). Sophisticated animal‐borne sensor systems, accelerometers in particular, now offer the possibility of continuously monitoring the activities of free‐ranging animals and their movement without the logistical difficulties of direct observation and accessibility (Fischer et al., 2018; Nathan et al., 2012; Shamoun‐Baranes et al., 2012; Wilson et al., 2006; Yoda et al., 2001). These tools have been applied in studies of many species and habitat types to answer questions on foraging and hunting (Hernández‐Pliego et al., 2017; Sato et al., 2015; Williams et al., 2015), energy expenditures (Collins et al., 2015; Elliott et al., 2014; Gómez Laich et al., 2011; Wilson et al., 2020), movement behavior (Ishii et al., 2017; Williams et al., 2020; Yoda et al., 2001), and migration strategy (Bishop et al., 2015; Weimerskirch et al., 2016). The diverse and expanding quality and quantity of accelerometer‐derived data have created a need for robust analytical methods to interpret patterns in data.

**FIGURE 1:**
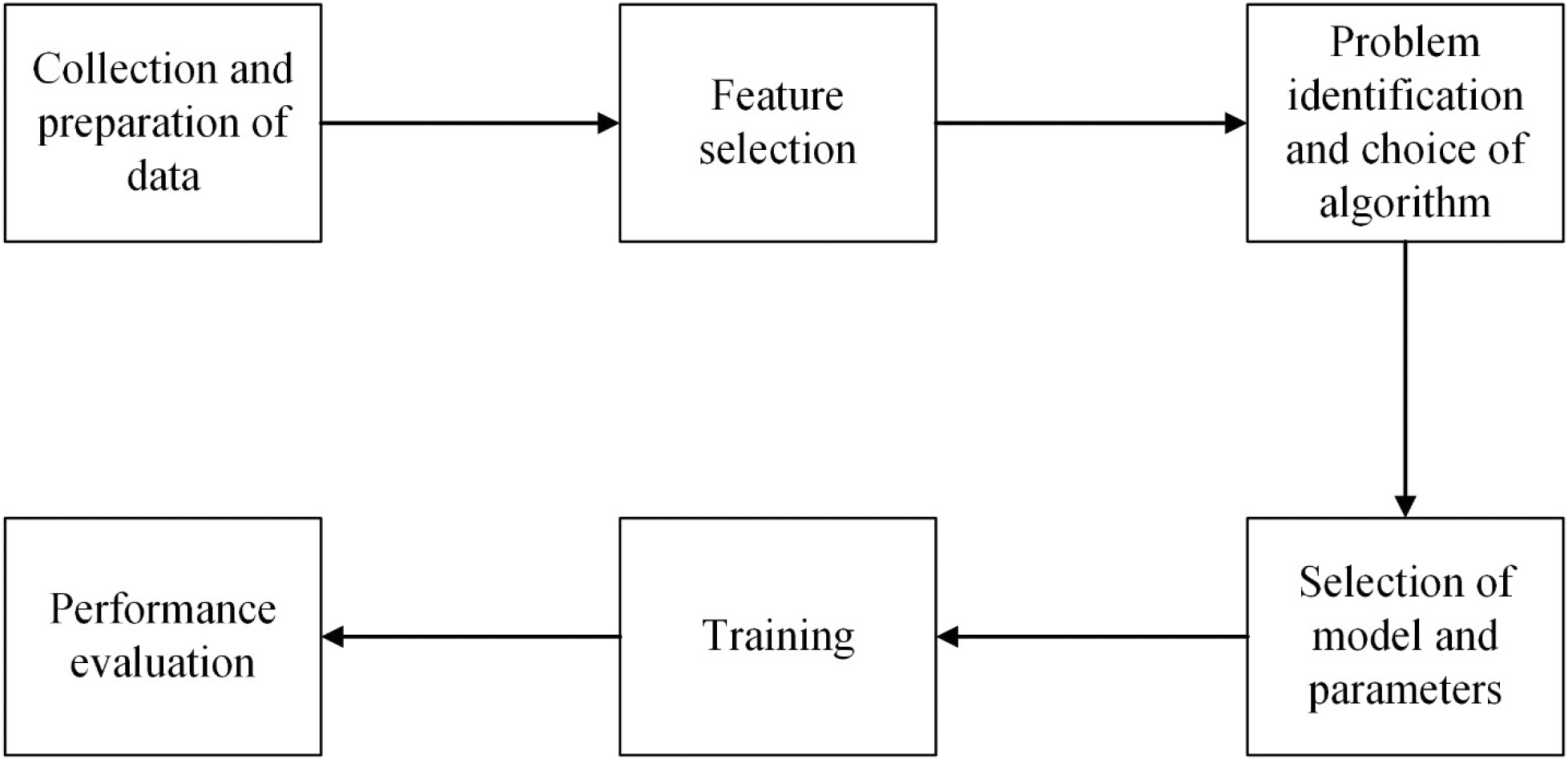
Representation of the components of a generic machine learning model. The steps shown in the figure were used to identify behavioral modes or states from accelerometry data in this study and are described in detail in Section 2.

A suite of methods has been developed to extract information about animal behavior from accelerometer data, many using techniques that have been broadly termed “machine learning”. The term “machine learning” (ML) has been defined as programming that allows a computer to learn from experience, where learning can be measured and scored iteratively (Samuel, 1959). Typically, machine learning involves a series of steps including data collection, feature selection, problem definition, algorithm and parameter selection, and model training and evaluation (Figure 1). For accelerometry, the simplest machine learning classification problems use validation data to inform the classification of tri‐axial measurement to behavior types (e.g., acceleration pattern x means behavior A and acceleration pattern y means behavior B; Studd et al., 2019). Without validation data, the ML classification problem can be approached from an unsupervised clustering perspective (Sakamoto et al., 2009). Alternatively, when only a small quantity of annotated acceleration data is available, it is possible to use a semi‐supervised ML method that blends elements of clustering and supervised learning (Tanha et al., 2012). Finally, machine learning research questions may focus on anomaly detection. In the case of accelerometry, this is less common, although anomalous readings are sometimes investigated to understand if they are driven by biology or technology (Tobin et al., 2020).

Unsupervised ML techniques commonly are used to interpret behavior from accelerometer data (Chimienti et al., 2016). Although fairly easy to implement, the accuracy of these methods is hard to interpret given that unsupervised methods do not require validation data. It is therefore surprising that there is little guidance as to the utility and appropriateness of unsupervised methods for these analyses. To address this problem, we evaluated the effectiveness of supervised (*n* = 6 modeling approaches) and semi‐supervised ML (*n* = 1) methods relative to that of two unsupervised methods to analyze accelerometry data. The data we used were collected from a critically endangered species—the California condor (*Gymnogyps californianus*)—that is the focus of extensive monitoring and management. Our analysis provides insight into the relative value of different approaches to the analysis of accelerometry data. We also use the information we generate to create a set of recommended best practices for interpreting accelerometry data.

## 2 MATERIALS AND METHODS

### 2.1 Study area and model species

The California condor is the largest soaring bird in North America. Under the framework of a “Condor Recovery Program,” condors are now captive‐bred and released to sustain a wild population spread across the southwestern United States and northwestern Mexico (USFWS, 2013). From approximately August to November of each year, captive‐bred juvenile condors are released at Bitter Creek National Wildlife Refuge, located in the foothills of the southwestern San Joaquin Valley in Kern County, California, USA (USFWS, 2013). Prior to release, the condors spend time in a captive enclosure (hereafter, a “flight pen”) with minimum exposure to humans (USFWS, 2013). This allows them to get familiar with the release site, and to engage in typical condor behaviors while interacting with wild condors that are perched or feeding nearby but outside the pen.

### 2.2 Accelerometry data collection

We outfitted nine condors in the flight pen with patagial tags, each with a unique ID, and a proprietary solar‐powered Global Positioning System‐Global System for Mobile Communications (GPS‐GSM) telemetry device weighing 50 g (Cellular Tracking Technologies, LLC). In addition to GPS data (which were not used in this study), the units collected tri‐axial acceleration data at a rate of 20 Hz. Although patagial tags were generally on the right wing, that was not always the case and there is a chance that differences among individuals could stem from tags being on different wings. Data were transmitted once daily over cellular networks and then downloaded to a server. For additional details on wing tagging and telemetry of condors, see Poessel et al. (2018).

### 2.3 Video data collection

We used digital cameras located inside the flight pen to record continuous video of condors and condor behavior. We recorded video continuously during daylight hours with two cameras (one AXIS P3367‐VE, Axis Communications AB and one AV3115, AV Costar) located at opposite ends of the flight pen. The cameras were mounted to the walls of the flight pen at ~2 m above ground in a configuration that together allowed observation of individual birds and identification of codes on patagial tags located at any spot within the flight pen. The digital data recorded by the cameras were backed up nightly to an external hard drive. We used the Milestone XProtect Essential+ application for video management and instantaneous viewing (Milestone Systems A/S).

### 2.4 Segmentation and identification of behaviors

Prior to classification, continuous accelerometry data usually are divided (hereafter, “segmented”) into either variable‐ or fixed‐time segments that use inherent characteristics within the data to identify boundaries (change points) between different behavioral states (Sur et al., 2017). We used variable time segments because this approach improves classification accuracy by better grouping together similar behaviors (Bom et al., 2014). We used a nonparametric model framework implemented with the function processStream in the package “cpm” (Ross, 2015) in R (v4.0.2; R Core Team, 2021) to identify change points (Bom et al., 2014; Shamoun‐Baranes et al., 2012; Sur et al., 2017). Because the mean and variance responded most strongly to accelerometer values on the *X* axis, these values were chosen as input to the model (e.g., Bom et al., 2014).

Once acceleration data were segmented, a single observer (MS) reviewed videos to assign behaviors from a pre‐defined ethogram to each of the variable‐time segments identified by the change point model. This pre‐defined ethogram was derived based on behaviors observed in the field and in preliminary viewing of the digital video we recorded. Our ethogram included eight types of behavior—sitting, walking, drinking, feeding, sunbathing, social interaction, preening, and flying (Table 1). Since the flight pens had snags on which condors perched, for those behaviors that could occur either on the ground or on a perch (sitting, walking, sunbathing, social interaction, and preening), we differentiated between events occurring on the ground versus those on a perch. If a segment included two behaviors, we categorized that segment with the behavior occupying the majority of time in the segment. Finally, we realized that some of these behaviors would be difficult to distinguish based solely on accelerometer data and that it would be difficult to identify behaviors on the ground versus on a perch. As such, we evaluated the initial performance of ML models after combining similar behaviors into a single category and ignoring ground versus perch categories, and we used in our models the reduced ethogram (see Section 3 for details).

**TABLE 1:** Ethogram of behaviors used to annotate video of behaviors of California condors.

Initial category	Final category	Occurs on		# segments (train/test)	Time (s)	Description
		Ground	Perch			
Walking	Walking	Y	Y	1318 (923/395)	850	Walking on the ground
Drinking	Drinking/Feeding	Y		3344 (2341/1003)	1775	Drinking at the water hole
Feeding	Drinking/Feeding	Y				Feeding on or walking around a carcass
Sitting	Sitting	Y	Y	148,136 (103,562/44,574)	95,948	Sitting on the ground
Sunbathing	Sitting	Y	Y			Sitting or walking, wings fully or partially outstretched
Social interaction	Sitting	Y	Y			Interaction with other birds inside or outside pen
Preening	Sitting	Y	Y			Preening behavior
Flying	Flying			149 (105/44)	123	Short flapping flights within the pen

*Note*: The initial ethogram included eight types of behavior—sitting, walking, drinking, feeding, sunbathing, social interaction, preening, and flying. The final category was the ethogram used in the analysis. Some behaviors occur only on the ground, others on the ground or a perch. The number of segments in total, as well as in the training and testing dataset, and the total time annotated into each behavior category are also reported (see Section 2.4 for more details). Finally, a short description of each behavior type is provided.

### 2.5 Accelerometer‐derived metrics

We considered 33 accelerometer‐derived metrics as input into our model (Brown et al., 2013; Nathan et al., 2012; Shamoun‐Baranes et al., 2012). Of these, 24 metrics were calculated using raw data from each of the three axes collected over a single segment of behavior that was defined by the change‐point model. On each axis, eight metrics were considered; these were the mean, standard deviation, median, minimum, maximum, range, skewness, and kurtosis. The remaining nine metrics were derived. These included the static and dynamic acceleration measured on each of the three axes (six metrics), and the pitch, overall dynamic body acceleration, and wing beat frequency (details of calculation methods for each of these are described in Patterson et al., 2018). All derived metrics were first calculated on the raw data and then averaged for each segment.

### 2.6 Data organization

We used the package “caret” in R (Kuhn et al., 2020; v: 6.0–86) to implement the model‐building and evaluation processes. Details of the functions that can be used for pre‐processing, data splitting, model tuning, and estimating model accuracy are provided in the resources for this package (Kuhn, 2019). Here, we review the subset of steps that we followed for this study. As such, these may provide a convenient guide to others who wish to build predictive machine learning models using accelerometer data. The steps we followed within “caret” are as follows:

1. *Pre‐processing*: We began with the process of “feature selection,” to reduce the set of accelerometer‐derived metrics used as input into our models (Nathan et al., 2012; Patterson et al., 2018). Feature selection improves the performance of machine learning algorithms and reduces the computational time (Kuhn, 2008, 2012). We used the function “findCorrelation” within “caret” to create a correlation matrix and find and remove highly correlated accelerometer‐derived metrics (Yu & Klaassen, 2021). The function considers the absolute values of pairwise correlations and, when two variables are highly correlated, it removes the variable with the largest mean absolute correlation. We used a pairwise absolute cut‐off of 0.75 to identify “highly” correlated variables (Kuhn & Johnson, 2013).
2. *Data splitting*: We used 70% of our data for training the model (hereafter, the “training data”) and the remaining 30% to test classification accuracy (“testing data”). We used the function “createDataPartition” within “caret” to generate a balanced 70/30 split of the data (i.e., balanced meaning that the distribution of behavior types within the testing and training datasets mimicked that in the overall dataset; Nathan et al., 2012).
3. *Subsampling for class imbalance*: Our data were significantly unbalanced with a significant disparity of frequency of observed behaviors. In order to correct this, we used the synthetic minority oversampling technique or SMOTE from the package “DMwR” on the training data (function “SMOTE”; v 0.4.1, Torgo, 2011).
4. *Centering and scaling*: We centered and scaled the variables of the training dataset sequentially using the functions, “preProcess” and “predict. preProcess” within “caret”. The first of these determines which continuous variables need to be centered and scaled, and the second centers and scales those variables. This process can either be done as a stand‐alone step or incorporated into the subsequent training step.
5. *Cross‐validation and tuning*: The training dataset can also be used for evaluating the performance of the algorithms using cross‐validation and for tuning the parameters of the algorithms. Although most algorithms have default parameter settings, the performance of the algorithm can be improved for each individual study by changing the values of the parameters. Tuning is described below for each model type.

### 2.7 Supervised and semi‐supervised ML models

We trained and evaluated the accuracy of six supervised and one semi‐supervised ML model, all of which have previously been used to analyze accelerometer data from animal biotelemetry. The six supervised model types we considered were random forest (RF; Breiman, 2001; Dickinson et al., 2021), k‐nearest neighbor (kNN; Yang et al., 2021), classification and regression tree (CART; Jeantet et al., 2018), support vector machine (SVM; Yang et al., 2021), neural network (NN; Kadar et al., 2020), and linear discriminant analysis (LDA; Oczak et al., 2016).

We implemented models within the package “caret” and compared them using their resampling distribution (Eugster & Leisch, 2011; Hothorn et al., 2005). We used the “train” function in “caret” to automate the process of model training, parameter tuning, and model selection based on optimal values of these parameters. All models were fit on the same training data and using the same resampling profiles. We used the argument “trainControl” to specify the type of resampling (trainControl(method = “repeatedcv”, number 10, repeats = 3)) and the argument “allowParallel = TRUE” to allow parallel processing and reduce computational time. This approach produces resampling results across tuning parameters (indicated by accuracy and kappa statistics). We also tested the performance of the models on the testing data.

We also customized the tuning process for a subset of our supervised models using the function “tunelength,” with a value of 10, meaning that the performance of a model was evaluated using 10 different values of each tuning parameter. Since this is a computationally intensive task, we customized the tuning process for only the top two models and evaluated their performance on the testing data.

The one semi‐supervised model whose performance we evaluated was the nearest mean classifier from the package “RSSL” (v.0.9.5; Krijthe & Loog, 2015; Krijthe, 2016). Since the algorithm works by iteratively labeling the unlabeled behaviors and adding these predictions to the set of labeled behaviors until the classifier converges, we first randomly removed 70% of the labels from the training dataset using the function “add_missinglabels_mar.” We then used the function “SelfTraining” to predict the labels of the unlabeled data of the training dataset. Of these predicted class labels, the ones with the highest probability of being correct are adopted as “pseudo‐labels” and the algorithm is iteratively improved. These data with the labeled and pseudo‐labels were then used to train the final algorithm.

Finally, we used our best‐performing models of each model type, with model‐specific tuning parameters, to predict behavior types in the testing data (function “predict” in “caret”). We then used the function “confusionMatrix” to construct a confusion matrix of actual and predicted behavior to evaluate the performance of the algorithms.

### 2.8 Unsupervised ML approaches

We evaluated the performance of two unsupervised ML approaches, K‐means clustering (Sakamoto et al., 2009) and EM clustering (“expectation–maximization”; Chimienti et al., 2016). We first implemented these models on the training data, setting the model to identify an optimal number of clusters. We used the function “fviz_nbclust” to determine and visualize the optimal number of clusters using within‐cluster sums of squares (package “factoextra"; Kassambara & Mundt, 2017; v 1.0.7). We then evaluated descriptive statistics for each cluster to identify the behavior they represented. Finally, we used the observed clusters to classify the testing dataset.

We implemented K‐means using the function “KMeans_rcpp” from the package ClusterR (v. 1.2.4; Mouselimis, 2021). We ran the algorithm on the training data with five initializations and a maximum of 10 iterations (Mouselimis, 2021). The computational time of the algorithm can be adjusted using the number of initializations and iterations (num_init, max_iters; Mouselimis, 2021). The centroids identified from the training dataset were then used to cluster the testing dataset using the function “predict_KMeans.” Similarly, we used the function “MclustDA” from the package “mclust” (v. 5.4.7; Fraley & Raftery, 2002) to cluster the training data using the EM algorithm. We then used the “predict” function from the same package to cluster the testing dataset.

## 3 RESULTS

The full dataset we considered included 27.4 h of paired video and accelerometry data ranging from 0.6 to 5.9 h per condor (median = 3.08 h). This included 152,947 behavioral segments, of which <1% were of birds flying, <1% were of walking, 2.2% were of drinking or feeding, and 96.9% were of sitting (Table 1). There were 1807–53,280 segments per bird (median = 16,355). Length of segments ranged from 0 to 54 s and from 2 to 1075 accelerometer measurements. Fewer than 1% of unclassified segments had >1 behavior and none had >2. For the supervised classification models, our training dataset included 106,931 segments, and our testing dataset was the remaining 46,016 segments. Behaviors were proportionally divided among the training and testing datasets (Table 1).

After the initial evaluation of model performance, we verified that our ML models had difficulty distinguishing sitting from social interaction, sunbathing, and preening. We, therefore, grouped all four of these behaviors into a single “sitting” category. Likewise, models were unable to distinguish drinking from feeding, so we grouped these two behaviors together. This process resulted in a reduced ethogram with four behavioral classes—“Walking,” “Drinking/Feeding,” “Sitting,” and “Flying”—that were input into classification models.

**FIGURE 2:**
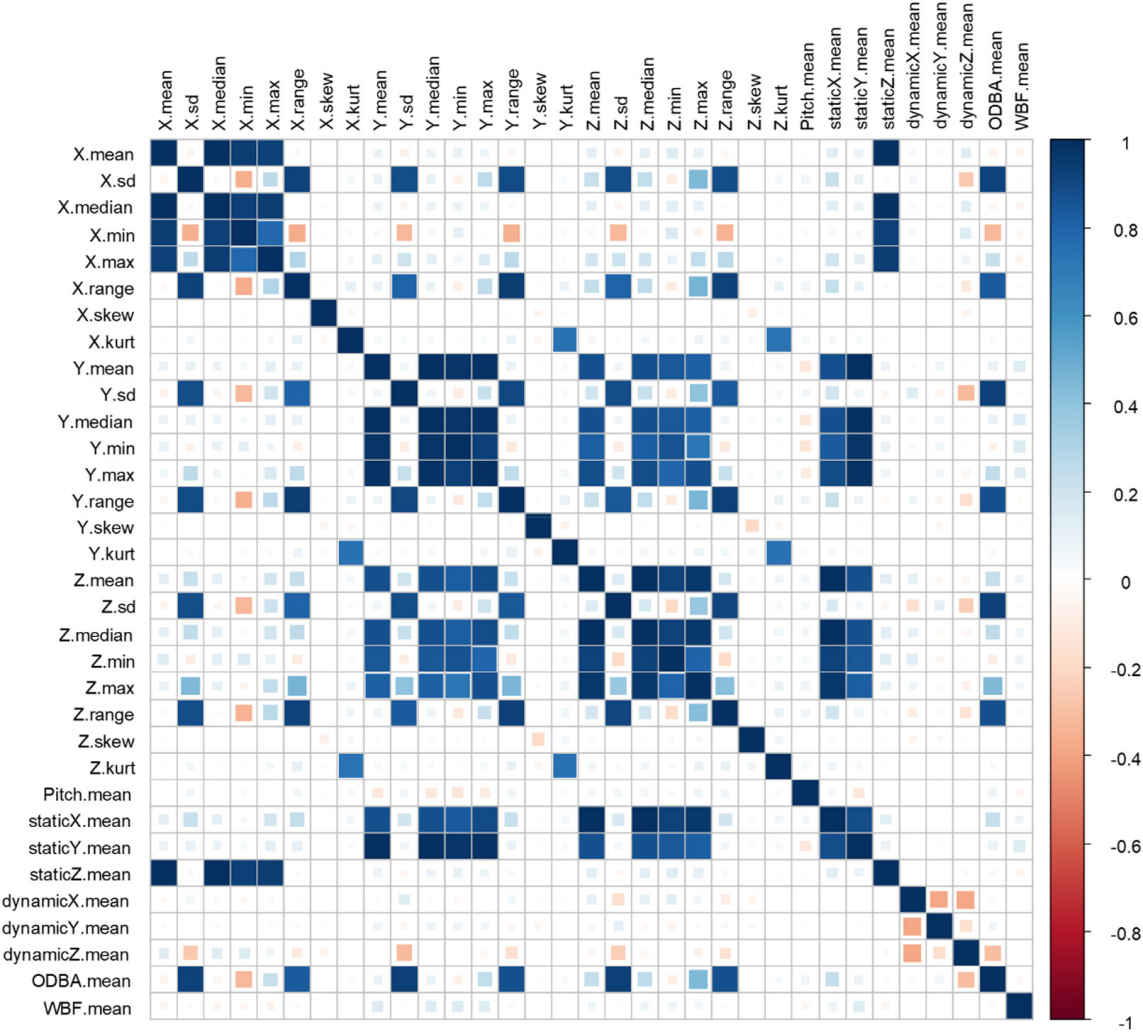
Pairwise correlation of 33 accelerometer‐derived metrics that were used as input into our model. Of these, 24 metrics were calculated using raw data from each of the three axes (*X*, *Y*, and *Z*) collected over a single change‐point model‐defined segment of behavior. These 24 metrics were eight statistics—the mean, standard deviation, median, minimum, maximum, range, skewness, and kurtosis—each calculated on each of the three axes. The remaining nine metrics were derived from these data and included the static and dynamic acceleration measured on each of the three axes, as well as the pitch, overall dynamic body acceleration (ODBA), and wing beat frequency (WBF). The figure shows the sign and magnitude of the correlation value, using two colored hues, where the intensity of color increases uniformly as the correlation value moves away from 0. Color (blue for positive values, red for negative values) signifies the sign of the correlation while the fill area is proportional to the absolute value of the correlation.

We detected a substantial correlation among accelerometer‐derived metrics (Figure 2) and ultimately removed 25 variables from consideration. The eight we retained included the median of the *Y* axis, standard deviation of the *Z* axis, mean of pitch, static acceleration on the *Z* axis, dynamic acceleration on the *X* axis, dynamic acceleration on the *Y* axis, dynamic acceleration on the *Z* axis and wing beat frequency (WBF). The distribution of these metrics among the four behaviors we considered is given in SI1 in Data S1.

### 3.1 Comparison of ML modeling approaches

Mean classification accuracy during training of the six supervised ML models we considered ranged from 0.59 to 0.91 (Table 2, SI2 in Data S1). Of these, random forest (RF) and k‐nearest neighbor (kNN) performed the best, each with overall predictive accuracies >0.81. Kappa statistics were also highest for RF and kNN, in most cases substantially greater than for other modeling approaches. Linear discriminant models were the worst performers of the models we considered, and neural network, support vector machine, and classification and regression tree were intermediate performers. Interestingly, when we applied these models to the testing data, the balanced accuracy when predicting all four behaviors was highest for the neural network (SI3 and SI4 in Data S1). Sensitivity and specificity of algorithms were variable, although generally reasonable for flying and sitting behaviors, and poor for drinking/feeding and walking (SI3 in Data S1).

**TABLE 2:** Model accuracy parameters for six supervised classification models of accelerometer data collected from California condors.

Model	Accuracy				Kappa			
	Minimum	Median	Mean	Maximum	Minimum	Median	Mean	Maximum
RF	0.82	0.92	0.91	0.99	0.66	0.84	0.83	0.97
kNN	0.73	0.81	0.81	0.96	0.47	0.63	0.64	0.92
NN	0.70	0.80	0.79	0.90	0.39	0.61	0.59	0.80
SVM	0.69	0.76	0.76	0.90	0.40	0.51	0.53	0.81
CART	0.57	0.68	0.69	0.82	0.16	0.37	0.38	0.64
LDA	0.51	0.58	0.59	0.69	0.00	0.14	0.17	0.38

*Note*: Models were ranked by two parameters, mean accuracy and mean Kappa. Model types are random forest (RF), k‐nearest neighbor (kNN), neural network (NN), support vector machine (SVM), classification and regression tree (CART), and linear discriminant analysis (LDA).

We customized the tuning parameters for the two supervised models with the best performance (random forest and k‐nearest neighbor). For RF, we evaluated model performance using values of *mtry* ranging from 1 to 10 (*mtry* is the number of variables randomly sampled at each split in the random forest algorithm). Accuracy of the algorithm was best with a *mtry* value of 5. Likewise, for kNN, we evaluated model performance using values of *k* ranging from 5 to 25 (*k* is the number of neighbors considered by the algorithm to determine the classification of a specific query point). Accuracy of the algorithm was best when the value of *k* was 7. Customizing the tuning process for these models did not change their performance, whether measured overall or by any one of the eight parameters we considered, including balanced accuracy (Table 3).

**TABLE 3:** Model accuracy parameters for two different approaches (random forest (RF) and k‐nearest neighbor (kNN)) to supervised classification of accelerometer data collected from California condors.

Parameter	RF				kNN			
	Drinking	Flying	Sitting	Walking	Drinking	Flying	Sitting	Walking
Sensitivity	0.00	0.68	0.76	0.00	0.02	0.74	0.71	0.00
Specificity	1.00	0.74	0.68	1.00	1.00	0.70	0.69	0.00
Pos. pred value	—	0.00	0.99	—	0.28	0.00	0.99	—
Neg. pred value	0.98	1.00	0.08	0.99	0.98	1.00	0.07	0.99
Prevalence	0.02	0.00	0.97	0.01	0.02	0.00	0.97	0.83
Detection rate	0.00	0.00	0.73	0.00	0.00	0.00	0.68	0.00
Detection Prevalence	0.00	0.26	0.74	0.00	0.00	0.31	0.69	0.00
Balanced accuracy	0.50	0.71	0.72	0.50	0.51	0.72	0.70	0.50

*Note*: Cells without numbers were incalculable for that parameter. Parameters are defined in S2 supporting information of Sur et al. (2017).

The semi‐supervised nearest mean classifier showed similar patterns in performance as did the supervised algorithms (Table 4). The overall accuracy of the algorithm was 0.61, and the balanced accuracy was highest for sitting and flying behaviors (0.65 in both cases), but poor for other behavior classes. Model classification parameters were generally worse for semi‐supervised nearest mean classifier than they were for RF and kNN models.

**TABLE 4:** Model accuracy parameters for a semi‐supervised nearest mean classification model applied to accelerometer data collected from California condors.

Parameter	Nearest mean classifier			
	Drinking	Flying	Sitting	Walking
Sensitivity	0.00	0.69	0.59	0.00
Specificity	0.97	0.62	0.71	0.99
Pos. pred value	0.00	0.59	0.69	0.00
Neg. pred value	0.97	0.71	0.61	0.99
Prevalence	0.03	0.44	0.52	0.01
Detection rate	0.00	0.30	0.31	0.00
Detection prevalence	0.03	0.52	0.45	0.01
Balanced accuracy	0.48	0.65	0.65	0.50

*Note*: Parameters are defined in S2 supporting information of Sur et al. (2017).

The optimal number of clusters for unsupervised modeling was four (SI5 in Data S1), and K‐means and EM clustering had overall classification accuracies of 0.61 and 0.77, respectively. However, the kappa statistics for both models were extremely poor at −0.02 to 0.06, respectively. Both unsupervised classification models, K‐means and EM clustering, resulted in class‐specific, balanced classification accuracies that generally were similar or worse than either the RF or kNN models (Table 5). That said, it is notable that these models performed reasonably well at predicting the two less prevalent behavior classes, drinking/feeding and walking.

**TABLE 5:** Model accuracy parameters for two approaches to unsupervised classification (K‐means clustering and expectation maximization (EM) clustering) of accelerometer data collected from California condors.

Parameter	K‐means clustering				EM clustering			
	Drinking	Flying	Sitting	Walking	Drinking	Flying	Sitting	Walking
Sensitivity	0.03	0.00	0.79	0.07	0.76	0.08	0.62	0.31
Specificity	0.86	1.00	0.13	0.93	0.84	0.97	0.77	0.82
Pos. pred value	0.00	0.00	0.97	0.01	0.09	0.00	0.99	0.01
Neg. pred value	0.98	1.00	0.02	0.99	0.99	1.00	0.06	0.99
Prevalence	0.02	0.00	0.97	0.01	0.02	0.00	0.97	0.01
Detection rate	0.00	0.00	0.76	0.00	0.02	0.00	0.60	0.00
Detection prevalence	0.13	0.00	0.79	0.07	0.18	0.03	0.60	0.18
Balanced accuracy	0.45	0.50	0.46	0.50	0.80	0.52	0.70	0.57

*Note*: Parameters are defined in S2 supporting information of Sur et al. (2017).

## 4 DISCUSSION

Here, we have demonstrated the importance and value of supervised classification approaches to the analysis of accelerometry data. Unsupervised approaches for the classification of behavior, although commonly used (Bishop et al., 2015; Chimienti et al., 2016; Sakamoto et al., 2009), provided some unique information, but overall they lacked the accuracy, and subsequent value for inference, of supervised approaches. Furthermore, although there have recently been several studies comparing different types of supervised classification methods (Rast et al., 2020; Tatler et al., 2018; Yu et al., 2021), our study is one of few (e.g., Patterson et al., 2018) that specifically compares unsupervised, semi‐supervised, and supervised approaches to classification. As such, this study provides important insight into not only the relative value of these different approaches, but into when it may be appropriate to use each type of approach.

### 4.1 Comparison of ML modeling approaches

There were distinct differences in performance not only among supervised, semi‐supervised, and unsupervised approaches, but also among the six supervised modeling approaches we evaluated. Our analysis provides no support for the continued use of unsupervised ML approaches to the classification of a priori‐determined behavioral classes and little support for the semi‐supervised nearest mean classification approach. Furthermore, among the supervised approaches, RF and kNN performed best (as indicated by kappa statistics), suggesting that these two approaches may be best suited to future classification problems. The existing literature is fairly consistent with these findings. Specifically, both Tatler et al. (2018) and Yu et al. (2021) found that RF approaches were among the most effective at classifying accelerometry data, and the latter also noted that LDA performed poorly. Both these studies suggested that other approaches (naïve Bayes, SVM, kNN in Tatler et al., 2018; SVM, artificial NN, and XGBoost in Yu et al., 2021) could also perform well, although the details of which approach performed best seemed to vary with the situation and the species under consideration. That said, neither of these two prior studies nor any of the others we found, evaluated the full suite of accuracy statistics we considered here.

### 4.2 Inference from unsupervised classification of bio‐logging data

There is a growing trend in the classification literature calling for or using either supervised classification or post hoc calibration of behavior when conducting analysis of accelerometry or other biologging data (Elbroch et al., 2018; Garrod et al., 2021; Halsey, 2017; Halsey & Bryce, 2021; Rast et al., 2020; Van Walsum et al., 2020). The body of literature in this field is growing more robust as there is an increase in the diversity of species evaluated and analytical techniques considered. Furthermore, these analyses show that if the goal is interpreting overall energy expenditure, specific behaviors that are defined a priori, or specific aspects of behavior (e.g., characteristics of wing beats or strides), unsupervised models are a poor choice (although, as we discuss below, there is a role for these approaches).

As one of the first papers whose explicit goal was specifically to compare supervised versus other approaches to classification, our study is consistent with other studies that call into question some uses of unsupervised classification of bio‐logging data. It is true that there can be substantial difficulties required to gather supervised data or to validate or calibrate classification. Despite these challenges, it is increasingly recognized that this effort is essential for inference drawn from matching of a priori‐defined behaviors to accelerometry data.

Despite the weaknesses of unsupervised classification approaches, there are situations where using this toolkit is appropriate. EM clustering in particular performed reasonably well at identifying rarely encountered behaviors. Likewise, although unsupervised classification is a poor choice for the identification of a priori‐defined behaviors, it can effectively identify patterns or clusters in accelerometer signals, and these can be interpreted as “behavioral modes.” As such, it is reasonable to use post hoc data interpretation to characterize predominant behaviors within those modes. For example, K‐means clustering has been used to identify behavioral “groups” from accelerometer data collected from European shag (*Phalacrocorax aristotelis*) in Scotland, UK (Sakamoto et al., 2009), and “movement states” from short‐interval GPS data collected from bald eagles in the midwestern USA (Bergen et al., 2022). In situations such as these, use of unsupervised classification is appropriate, since inference is not to specific behaviors, but to “modes” or “states” in which accelerometer data cluster together.

## 5 CONCLUSIONS

Our work, together with that from earlier studies, suggests several best practices for use of accelerometry data to draw inference about animal behavior. These are:

1. Unsupervised classification techniques are not appropriate for identifying a priori‐defined behaviors in telemetry data. These tools are reasonable for information gathering and inference to post hoc definition of more generalized behavioral modes or states.
2. Those wishing to use ML tools to identify behavioral modes or states from accelerometry data may wish to evaluate several ML classification algorithms to identify which works best with the unique features of their data. This is likely especially important when behavioral datasets are unbalanced, as was ours and as would be most biologically realistic data.
3. In general, RF and kNN were consistently effective at classifying our data. SVM, NN, and perhaps others (XGBoost and naïve Bayes) could also be evaluated for other datasets.
4. Use of a single metric of accuracy is unlikely to convey the full story about model performance. In this study, and in others noted above, overall model accuracies tended to be high for all supervised classification approaches. However, a more detailed evaluation of other relevant statistics (e.g., Tables 2 and 3) illustrates substantial, nuanced, and highly informative differences among model types.

## AUTHOR CONTRIBUTIONS

**Maitreyi Sur:** Conceptualization (equal); formal analysis (equal); investigation (equal); methodology (equal); writing – original draft (equal); writing – review and editing (equal). **Jonathan C. Hall:** Conceptualization (equal); funding acquisition (equal); writing – review and editing (equal). **Joseph Brandt:** Data curation (equal); writing – review and editing (equal). **Molly Astell:** Data curation (equal); writing – review and editing (equal). **Sharon A. Poessel:** Conceptualization (equal); writing – review and editing (equal). **Todd E. Katzner:** Conceptualization (equal); funding acquisition (equal); methodology (equal); writing – original draft (equal); writing – review and editing (equal).

## CONFLICT OF INTEREST STATEMENT

The authors declare no competing interests.

## Supporting information


Data S1:

## Data Availability

The data used for this study are available in a USGS Data Release at https://doi.org/10.5066/P9PAVUEZ.

## References

[ece310035-bib-0001] Bergen, S. , Huso, M. M. , Duerr, A. E. , Braham, M. A. , Katzner, T. E. , Schmuecker, S. , & Miller, T. A. (2022). Classifying behavior from short‐interval biologging data: An example with GPS tracking of birds. Ecology and Evolution, 12(2), e08395. 35154643 10.1002/ece3.8395 PMC8819645

[ece310035-bib-0002] Bishop, C. M. , Spivey, R. J. , Hawkes, L. A. , Batbayar, N. , Chua, B. , Frappell, P. B. , Milsom, W. K. , Natsagdorj, T. , Newman, S. H. , Scott, G. R. , Takekawa, J. Y. , Wikelski, M. , & Butler, P. J. (2015). The roller coaster flight strategy of bar‐headed geese conserves energy during Himalayan migrations. Science, 347(6219), 250–254. 25593180 10.1126/science.1258732

[ece310035-bib-0003] Bom, R. A. , Bouten, W. , Piersma, T. , Oosterbeek, K. , & van Gils, J. A. (2014). Optimizing acceleration‐based ethograms: The use of variable‐time versus fixed‐time segmentation. Movement Ecology, 2(1), 6. 25520816 10.1186/2051-3933-2-6 PMC4267607

[ece310035-bib-0004] Breiman, L. (2001). Random forests. Machine Learning, 45(1), 5–32.

[ece310035-bib-0005] Brown, D. D. , Kays, R. , Wikelski, M. , Wilson, R. , & Klimley, A. (2013). Observing the unwatchable through acceleration logging of animal behavior. Animal Biotelemetry, 1(1), 20.

[ece310035-bib-0006] Chimienti, M. , Cornulier, T. , Owen, E. , Bolton, M. , Davies, I. M. , Travis, J. M. , & Scott, B. E. (2016). The use of an unsupervised learning approach for characterizing latent behaviors in accelerometer data. Ecology and Evolution, 6(3), 727–741. 26865961 10.1002/ece3.1914 PMC4739568

[ece310035-bib-0007] Collins, P. M. , Green, J. A. , Warwick‐Evans, V. , Dodd, S. , Shaw, P. J. A. , Arnould, J. P. Y. , & Halsey, L. G. (2015). Interpreting behaviors from accelerometry: A method combining simplicity and objectivity. Ecology and Evolution, 5(20), 4642–4654. 26668729 10.1002/ece3.1660 PMC4670056

[ece310035-bib-0008] Dickinson, E. R. , Twining, J. P. , Wilson, R. , Stephens, P. A. , Westander, J. , Marks, N. , & Scantlebury, D. M. (2021). Limitations of using surrogates for behaviour classification of accelerometer data: Refining methods using random forest models in Caprids. Movement Ecology, 9(1), 1–14. 34099067 10.1186/s40462-021-00265-7 PMC8186069

[ece310035-bib-0009] Elbroch, L. M. , Lowrey, B. , & Wittmer, H. U. (2018). The importance of fieldwork over predictive modeling in quantifying predation events of carnivores marked with GPS technology. Journal of Mammalogy, 99(1), 223–232.

[ece310035-bib-0010] Elliott, K. H. , Chivers, L. S. , Bessey, L. , Gaston, A. J. , Hatch, S. A. , Kato, A. , Osborne, O. , Ropert‐Coudert, Y. , Speakman, J. R. , & Hare, J. F. (2014). Windscapes shape seabird instantaneous energy costs but adult behavior buffers impact on offspring. Movement Ecology, 2(1), 1–15. 26019870 10.1186/s40462-014-0017-2 PMC4445632

[ece310035-bib-0011] Eugster, M. J. , & Leisch, F. (2011). Exploratory analysis of benchmark experiments an interactive approach. Computational Statistics, 26, 699–710.

[ece310035-bib-0012] Fischer, M. , Parkins, K. , Maizels, K. , Sutherland, D. R. , Allan, B. M. , Coulson, G. , & di Stefano, J. (2018). Biotelemetry marches on: A cost‐effective GPS device for monitoring terrestrial wildlife. PLoS One, 13(7), e0199617. 30063710 10.1371/journal.pone.0199617 PMC6067714

[ece310035-bib-0013] Fraley, C. , & Raftery, A. E. (2002). MCLUST: Software for model‐based clustering, density estimation and discriminant analysis. University of Washington, Seattle, Department of Statistics.

[ece310035-bib-0014] Garrod, A. , Yamamoto, S. , Sakamoto, K. Q. , & Sato, K. (2021). Video and acceleration records of streaked shearwaters allows detection of two foraging behaviours associated with large marine predators. PLoS One, 16(7), e0254454. 34270571 10.1371/journal.pone.0254454 PMC8284635

[ece310035-bib-0015] Gómez Laich, A. , Wilson, R. P. , Gleiss, A. C. , Shepard, E. L. C. , & Quintana, F. (2011). Use of overall dynamic body acceleration for estimating energy expenditure in cormorants. Does locomotion in different media affect relationships? Journal of Experimental Marine Biology and Ecology, 399(2), 151–155.

[ece310035-bib-0016] Halsey, L. G. (2017). Relationships grow with time: A note of caution about energy expenditure‐proxy correlations, focussing on accelerometry as an example. Functional Ecology, 31(6), 1176–1183.

[ece310035-bib-0017] Halsey, L. G. , & Bryce, C. M. (2021). Proxy problems: Why a calibration is essential for interpreting quantified changes in energy expenditure from biologging data. Functional Ecology, 35(3), 627–634.

[ece310035-bib-0018] Hernández‐Pliego, J. , Rodríguez, C. , Dell'Omo, G. , & Bustamante, J. (2017). Combined use of tri‐axial accelerometers and GPS reveals the flexible foraging strategy of a bird in relation to weather conditions. PLoS One, 12(6), e0177892. 28591181 10.1371/journal.pone.0177892 PMC5462363

[ece310035-bib-0019] Hothorn, T. , Leisch, F. , Zeileis, A. , & Hornik, K. (2005). The design and analysis of benchmark experiments. Journal of Computational and Graphical Statistics, 14(3), 675–699.

[ece310035-bib-0020] Ishii, M. , Murase, H. , Fukuda, Y. , Sawada, K. , Sasakura, T. , Tamura, T. , Bando, T. , Matsuoka, K. , Shinohara, A. , Nakatsuka, S. , Katsumata, N. , Okazaki, M. , Miyashita, K. , & Mitani, Y. (2017). Diving behavior of sei whales *Balaenoptera borealis* relative to the vertical distribution of their potential prey. Mammal Study, 42(4), 1–9.

[ece310035-bib-0021] Jeantet, L. , Dell'Amico, F. , Forin‐Wiart, M. A. , Coutant, M. , Bonola, M. , Etienne, D. , Gresser, J. , Regis, S. , Lecerf, N. , Lefebvre, F. , & de Thoisy, B. (2018). Combined use of two supervised learning algorithms to model sea turtle behaviours from tri‐axial acceleration data. Journal of Experimental Biology, 221(10), jeb177378. 29661804 10.1242/jeb.177378

[ece310035-bib-0022] Kadar, J. P. , Ladds, M. A. , Day, J. , Lyall, B. , & Brown, C. (2020). Assessment of machine learning models to identify Port Jackson shark behaviours using tri‐axial accelerometers. Sensors, 20(24), 7096. 33322308 10.3390/s20247096 PMC7763149

[ece310035-bib-0023] Kassambara, A. , & Mundt, F. (2017). Package ‘factoextra’. Extract and visualize the results of multivariate data analyses, 76(2).

[ece310035-bib-0024] Kays, R. , Crofoot, M. C. , Jetz, W. , & Wikelski, M. (2015). Terrestrial animal tracking as an eye on life and planet. Science, 348(6240), aaa2478. 26068858 10.1126/science.aaa2478

[ece310035-bib-0025] Krijthe, J. H. (2016). RSSL: R package for semi‐supervised learning. In B. Kerautret , M. Colom , & P. Monasse (Eds.), Reproducible research in pattern recognition. RRPR 2016. Lecture Notes in Computer Science (Vol. 10214, pp. 104–115). Springer International Publishing.

[ece310035-bib-0026] Krijthe, J. H. , & Loog, M. (2015). Implicitly constrained semi‐supervised least squares classification. In E. Fromont , T. de Bie , & M. van Leeuwen (Eds.), 14th international symposium on advances in intelligent data analysis XIV (Lecture Notes in Computer Science) (Vol. 9385, pp. 158–169). Saint Etienne.

[ece310035-bib-0027] Kuhn, M. (2008). Building predictive models in R using the caret package. Journal of Statistical Software, 28(1), 1–26.27774042

[ece310035-bib-0028] Kuhn, M. (2012). Variable selection using the caret package . Retrieved from https://r‐forge.r‐project.org/scm/viewvc.php/*checkout*/pkg/caret/inst/doc/caretSelection.pdf?revision=77&root=caret&pathrev=90

[ece310035-bib-0029] Kuhn, M. (2019). The caret package . Retrieved February 4, 2022, from https://topepo.github.io/caret/index.html

[ece310035-bib-0030] Kuhn, M. , & Johnson, K. (2013). Applied predictive modeling (Vol. 26, pp. 27–59). Springer.

[ece310035-bib-0031] Kuhn, M. , Wing, J. , Weston, S. , Williams, A. , Keefer, C. , Engelhardt, A. , Cooper, T. , Mayer, Z. , Kenkel, B. , & R Core Team . (2020). Package ‘caret’. The R Journal, 223, 7.

[ece310035-bib-0033] Mouselimis, L. (2021). ClusterR: Gaussian mixture models, K‐means, mini‐batch‐Kmeans, K‐Medoids and affinity propagation clustering . R Package Version 1.2.5.

[ece310035-bib-0034] Nathan, R. , Spiegel, O. , Fortmann‐Roe, S. , Harel, R. , Wikelski, M. , & Getz, W. M. (2012). Using tri‐axial acceleration data to identify behavioral modes of free‐ranging animals: General concepts and tools illustrated for griffon vultures. The Journal of Experimental Biology, 215(6), 986–996. 22357592 10.1242/jeb.058602 PMC3284320

[ece310035-bib-0035] Oczak, M. , Maschat, K. , Berckmans, D. , Vranken, E. , & Baumgartner, J. (2016). Can an automated labelling method based on accelerometer data replace a human labeller? – Postural profile of farrowing sows. Computers and Electronics in Agriculture, 127, 168–175.

[ece310035-bib-0036] Patterson, A. , Grant Gilchrist, H. , Chivers, L. , Hatch, S. , & Elliott, K. (2018). A comparison of techniques for classifying behavior from accelerometers for two species of seabird. Ecology and Evolution, 9, 3030–3045. 10.1002/ece3.4740 PMC6434605 30962879

[ece310035-bib-0037] Poessel, S. A. , Brandt, J. , Mendenhall, L. , Braham, M. A. , Lanzone, M. J. , McGann, A. J. , & Katzner, T. E. (2018). Flight response to spatial and temporal correlates informs risk from wind turbines to the California condor. The Condor: Ornithological Applications, 120(2), 330–342.

[ece310035-bib-0038] R Core Team . (2021). R: A language and environment for statistical computing. R Foundation for Statistical Computing.

[ece310035-bib-0039] Rast, W. , Kimmig, S. E. , Giese, L. , & Berger, A. (2020). Machine learning goes wild: Using data from captive individuals to infer wildlife behaviours. PLoS One, 15(5), e0227317. 32369485 10.1371/journal.pone.0227317 PMC7200095

[ece310035-bib-0040] Ross, G. J. (2015). Parametric and nonparametric sequential change detection in R: The cpm package. Journal of Statistical Software, 66, 1–20.

[ece310035-bib-0041] Sakamoto, K. Q. , Sato, K. , Ishizuka, M. , Watanuki, Y. , Takahashi, A. , Daunt, F. , & Wanless, S. (2009). Can ethograms be automatically generated using body acceleration data from free‐ranging birds? PLoS One, 4, e5379. 19404389 10.1371/journal.pone.0005379 PMC2671159

[ece310035-bib-0042] Samuel, A. L. (1959). Some studies in machine learning using the game of checkers. IBM Journal of Research and Development, 3(3), 210–229.

[ece310035-bib-0043] Sato, N. N. , Kokubun, N. , Yamamoto, T. , Watanuki, Y. , Kitaysky, A. S. , & Takahashi, A. (2015). The jellyfish buffet: Jellyfish enhance seabird foraging opportunities by concentrating prey. Biology Letters, 11(8), 20150358. 26311157 10.1098/rsbl.2015.0358 PMC4571674

[ece310035-bib-0044] Shamoun‐Baranes, J. , Bom, R. , van Loon, E. E. , Ens, B. J. , Oosterbeek, K. , & Bouten, W. (2012). From sensor data to animal behaviour: An oystercatcher example. PLoS One, 7(5), e37997. 22693586 10.1371/journal.pone.0037997 PMC3365100

[ece310035-bib-0047] Studd, E. K. , Landry‐Cuerrier, M. , Menzies, A. K. , Boutin, S. , McAdam, A. G. , Lane, J. E. , & Humphries, M. M. (2019). Behavioral classification of low‐frequency acceleration and temperature data from a free‐ranging small mammal. Ecology and Evolution, 9(1), 619–630. 30680142 10.1002/ece3.4786 PMC6342100

[ece310035-bib-0048] Sur, M. , Suffredini, T. , Wessells, S. M. , Bloom, P. H. , Lanzone, M. , Blackshire, S. , Sridhar, S. , & Katzner, T. (2017). Improved supervised classification of accelerometry data to distinguish behaviors of soaring birds. PLoS One, 12(4), e0174785. 28403159 10.1371/journal.pone.0174785 PMC5389810

[ece310035-bib-0049] Tanha, J. , Van Someren, M. , de Bakker, M. , Bouteny, W. , Shamoun‐Baranes, J. , & Afsarmanesh, H. (2012). Multiclass semi‐supervised learning for animal behavior recognition from accelerometer data. 2012 IEEE 24th International Conference on Tools with Artificial Intelligence, Athens, Greece (pp. 690–697).

[ece310035-bib-0050] Tatler, J. , Cassey, P. , & Prowse, T. A. (2018). High accuracy at low frequency: Detailed behavioural classification from accelerometer data. Journal of Experimental Biology, 221(23), jeb184085. 30322979 10.1242/jeb.184085

[ece310035-bib-0051] Tobin, C. , Bailey, D. W. , Trotter, M. G. , & O'Connor, L. (2020). Sensor based disease detection: A case study using accelerometers to recognize symptoms of Bovine Ephemeral Fever. Computers and Electronics in Agriculture, 175, 105605.

[ece310035-bib-0052] Torgo, L. (2011). Data mining with R: Learning with case studies. Chapman and Hall/CRC.

[ece310035-bib-0053] U.S. Fish and Wildlife Service . (2013). Hopper Mountain, Bitter Creek, and Blue Ridge National Wildlife Refuges: Final comprehensive conservation plan and environmental assessment. USFWS, Pacific Southwest Region, Refuge Conservation Planning Branch.

[ece310035-bib-0054] Van Walsum, T. A. , Perna, A. , Bishop, C. M. , Murn, C. P. , Collins, P. M. , Wilson, R. P. , & Halsey, L. G. (2020). Exploring the relationship between flapping behaviour and accelerometer signal during ascending flight, and a new approach to calibration. Ibis, 162(1), 13–26.

[ece310035-bib-0056] Weimerskirch, H. , Bishop, C. , Jeanniard‐du‐Dot, T. , Prudor, A. , & Sachs, G. (2016). Frigate birds track atmospheric conditions over months‐long transoceanic flights. Science, 353(6294), 74–78. 27365448 10.1126/science.aaf4374

[ece310035-bib-0057] Williams, H. J. , Shepard, E. L. C. , Duriez, O. , & Lambertucci, S. A. (2015). Can accelerometry be used to distinguish between flight types in soaring birds? Animal Biotelemetry, 3(1), 45.

[ece310035-bib-0058] Williams, H. J. , Shepard, E. L. C. , Holton, M. D. , Alarcón, P. A. E. , Wilson, R. P. , & Lambertucci, S. A. (2020). Physical limits of flight performance in the heaviest soaring bird. Proceedings of the National Academy of Sciences of the United States of America, 117(30), 17884–17890. 32661147 10.1073/pnas.1907360117 PMC7395523

[ece310035-bib-0059] Wilson, R. P. , Börger, L. , Holton, M. D. , Scantlebury, D. M. , Gómez‐Laich, A. , Quintana, F. , Rosell, F. , Graf, P. M. , Williams, H. , Gunner, R. , Hopkins, L. , Marks, N. , Geraldi, N. R. , Duarte, C. M. , Scott, R. , Strano, M. S. , Robotka, H. , Eizaguirre, C. , Fahlman, A. , & Shepard, E. L. C. (2020). Estimates for energy expenditure in free‐living animals using acceleration proxies: A reappraisal. Journal of Animal Ecology, 89(1), 161–172. 31173339 10.1111/1365-2656.13040 PMC7030956

[ece310035-bib-0060] Wilson, R. P. , White, C. R. , Quintana, F. , Halsey, L. G. , Liebsch, N. , Martin, G. R. , & Butler, P. J. (2006). Moving towards acceleration for estimates of activity‐specific metabolic rate in free‐living animals: The case of the cormorant. Journal of Animal Ecology, 75(5), 1081–1090. 16922843 10.1111/j.1365-2656.2006.01127.x

[ece310035-bib-0061] Yang, X. , Zhao, Y. , Street, G. M. , Huang, Y. , To, S. F. , & Purswell, J. L. (2021). Classification of broiler behaviours using triaxial accelerometer and machine learning. Animal, 15(7), 100269. 34102430 10.1016/j.animal.2021.100269

[ece310035-bib-0062] Yoda, K. , Naito, Y. , Sato, K. , Takahashi, A. , & Nishikawa, J. (2001). A new technique for monitoring the behaviour of free‐ranging Adelie penguins. The Journal of Experimental Biology, 204, 685–690. 11171350 10.1242/jeb.204.4.685

[ece310035-bib-0063] Yu, H. , Deng, J. , Nathan, R. , Kröschel, M. , Pekarsky, S. , Li, G. , & Klaassen, M. (2021). An evaluation of machine learning classifiers for next‐generation, continuous‐ethogram smart trackers. Movement Ecology, 9(1), 1–14. 33785056 10.1186/s40462-021-00245-x PMC8011142

[ece310035-bib-0064] Yu, H. , & Klaassen, M. (2021). R package for animal behavior classification from accelerometer data—Rabc. Ecology and Evolution, 11(18), 12364–12377. 34594505 10.1002/ece3.7937 PMC8462134

