# Supplementary material for: Supervised versus unsupervised approaches to classification of accelerometry data
Source: Ecol Evol. 2023 May 17;13(5):e10035. doi: 10.1002/ece3.10035 (PMC10191777; doi:10.1002/ece3.10035)

**SUPPORTING INFORMATION**

SI1: Boxplots showing distribution of calculated metrics derived from accelerometer data collected from California condors. We considered four types of behavior - drinking and feeding, flying, sitting and walking. Metrics are median and mean dynamic acceleration of the Y axis; mean dynamic acceleration, mean static acceleration, and standard deviation of the Z axis; mean dynamic acceleration of the X axis; mean pitch; and mean wing beat frequency (see text for more details).


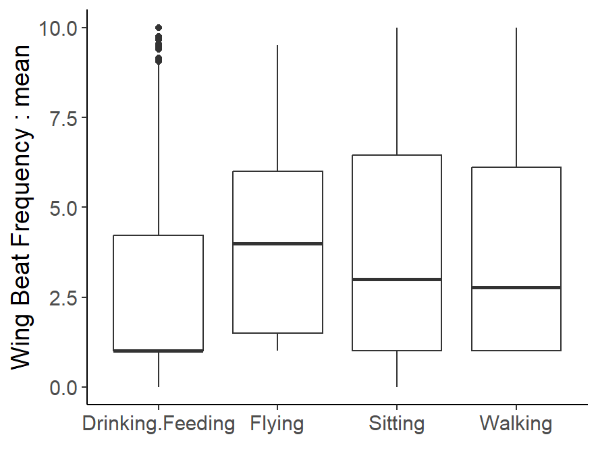

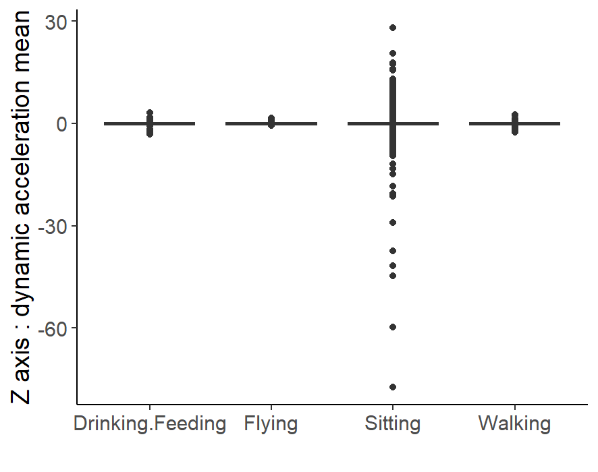

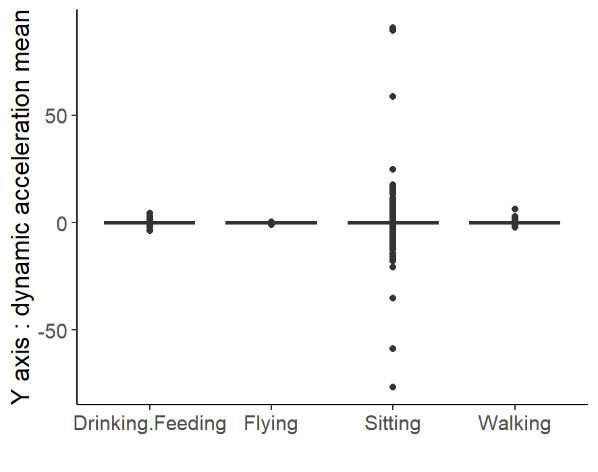

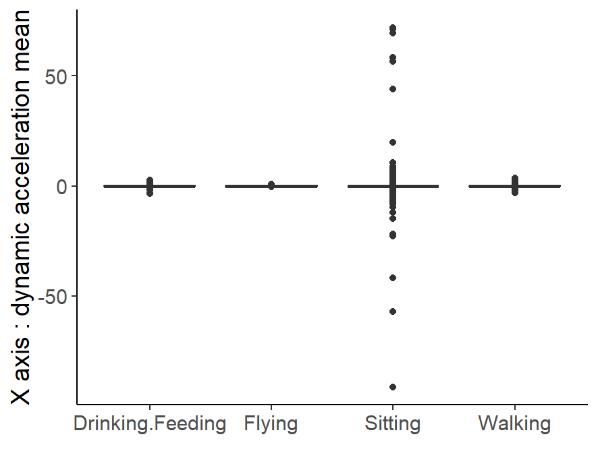

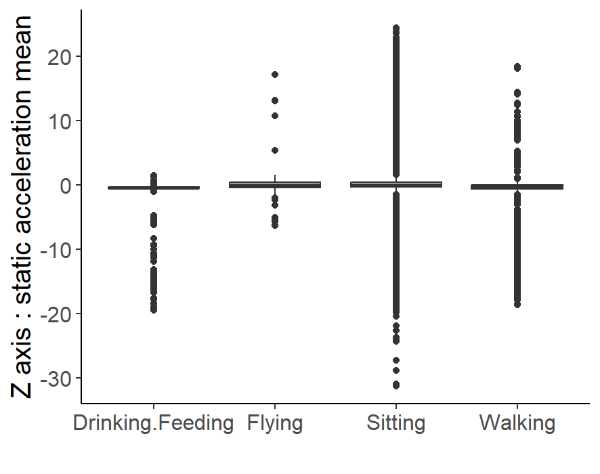

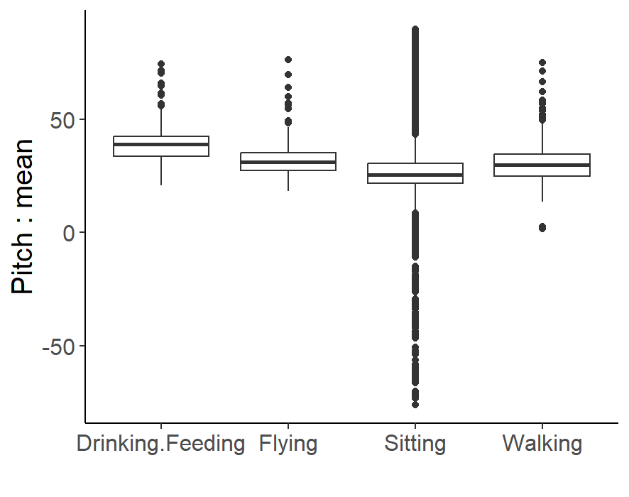

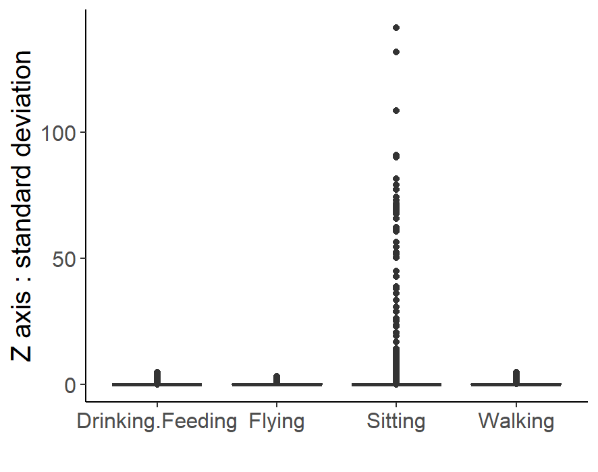

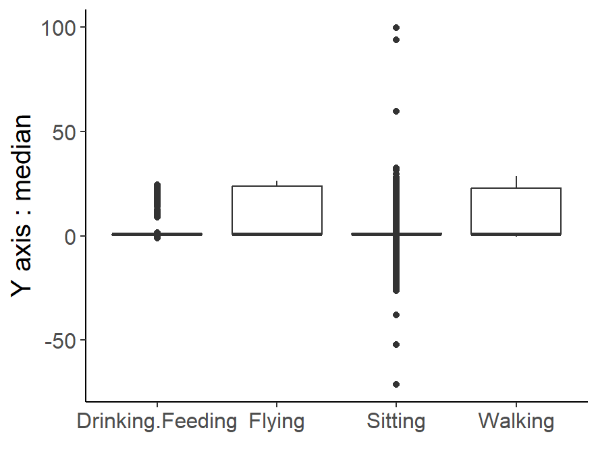


SI2: Performance metrics (Accuracy and Kappa) of 6 supervised model types: random forest (RF), k-nearest neighbor (KNN), neural network (NN), support vector machine (SVM), classification and regression tree (CART), and linear discriminant analysis (LDA). The performance metrics were calculated using cross validation (details in the text)


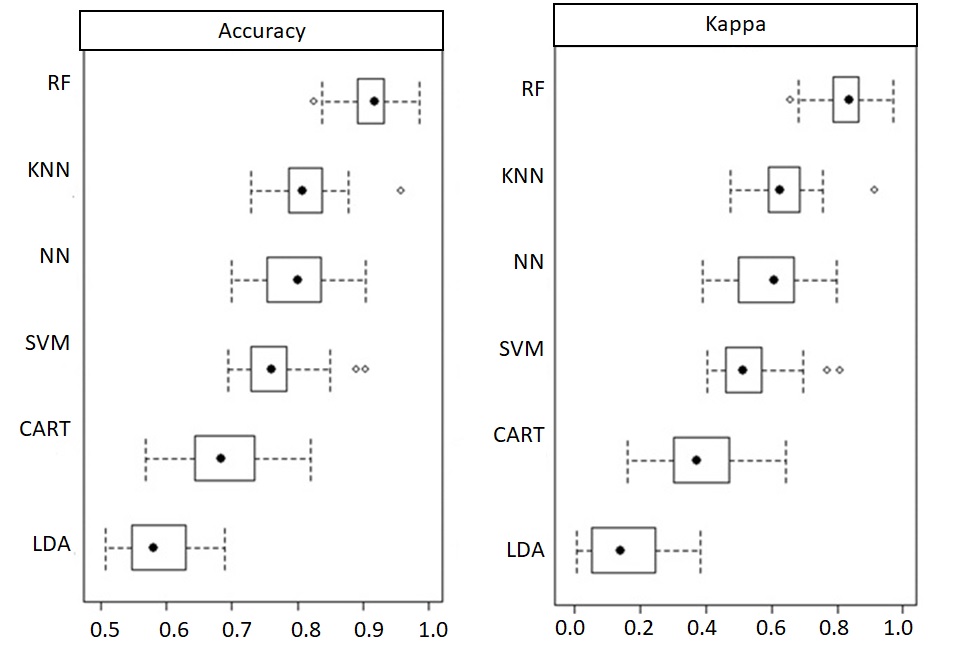


SI3: Model accuracy parameters of 6 different approaches to supervised classification of accelerometer data collected from California condors. Model types are random forest (RF), k-nearest neighbor (kNN), neural network (NN), support vector machine (SVM), classification and regression tree (CART), and linear discriminant analysis (LDA). Cells without numbers were incalculable for that parameter.

| Behavior | Parameter | RF | KNN | NN | SVM | CART | LDA |
| --- | --- | --- | --- | --- | --- | --- | --- |
| Drinking | Sensitivity | 0.00 | 0.02 | 0.00 | 0.00 | 0.00 | 0.00 |
|  | Specificity | 1.00 | 1.0 | 1.00 | 1.00 | 1.00 | 1.00 |
|  | Pos. Pred. Value | -- | 0.28 | -- | -- | -- | -- |
|  | Neg. Pred. Value | 0.98 | 0.98 | 0.98 | 0.98 | 0.98 | 0.98 |
|  | Prevalence | 0.02 | 0.02 | 0.02 | 0.02 | 0.02 | 0.02 |
|  | Detection Rate | 0.00 | 0.00 | 0.00 | 0.00 | 0.00 | 0.00 |
|  | Detection Prevalence | 0.00 | 0.00 | 0.00 | 0.00 | 0.00 | 0.00 |
|  | Balanced Accuracy | 0.50 | 0.51 | 0.50 | 0.50 | 0.50 | 0.50 |
|  |  |  |  |  |  |  |  |
| Flying | Sensitivity | 0.68 | 0.74 | 0.84 | 0.58 | 0.91 | 0.42 |
|  | Specificity | 0.74 | 0.70 | 0.68 | 0.78 | 0.47 | 0.76 |
|  | Pos. Pred. Value | 0.00 | 0.00 | 0.00 | 0.00 | 0.00 | 0.00 |
|  | Neg. Pred. Value | 1.00 | 1.00 | 1.00 | 1.00 | 1.00 | 1.00 |
|  | Prevalence | 0.00 | 0.00 | 0.00 | 0.00 | 0.00 | 0.00 |
|  | Detection Rate | 0.00 | 0.00 | 0.00 | 0.00 | 0.00 | 0.00 |
|  | Detection Prevalence | 0.26 | 0.30 | 0.32 | 0.22 | 0.53 | 0.24 |
|  | Balanced Accuracy | 0.71 | 0.72 | 0.76 | 0.68 | 0.69 | 0.59 |
|  |  |  |  |  |  |  |  |
| Sitting | Sensitivity | 0.76 | 0.71 | 0.70 | 0.79 | 0.48 | 0.76 |
|  | Specificity | 0.68 | 0.69 | 0.81 | 0.69 | 0.91 | 0.30 |
|  | Pos. Pred. Value | 0.99 | 0.99 | 0.99 | 0.99 | 0.99 | 0.97 |
|  | Neg. Pred. Value | 0.08 | 0.07 | 0.08 | 0.09 | 0.05 | 0.04 |
|  | Prevalence | 0.97 | 0.97 | 0.97 | 0.97 | 0.97 | 0.97 |
|  | Detection Rate | 0.73 | 0.68 | 0.68 | 0.77 | 0.47 | 0.74 |
|  | Detection Prevalence | 0.74 | 0.69 | 0.68 | 0.78 | 0.47 | 0.76 |
|  | Balanced Accuracy | 0.72 | 0.70 | 0.75 | 0.74 | 0.70 | 0.53 |
|  |  |  |  |  |  |  |  |
| Walking | Sensitivity | 0.00 | 0.00 | 0.00 | 0.00 | 0.00 | 0.00 |
|  | Specificity | 1.00 | 1.00 | 1.00 | 1.00 | 1.00 | 1.00 |
|  | Pos. Pred. Value | -- | -- | -- | -- | -- | 0.00 |
|  | Neg. Pred. Value | 0.99 | 0.99 | 0.99 | 0.99 | 0.99 | 0.99 |
|  | Prevalence | 0.01 | 0.01 | 0.01 | 0.01 | 0.01 | 0.01 |
|  | Detection Rate | 0.00 | 0.00 | 0.00 | 0.00 | 0.00 | 0.00 |
|  | Detection Prevalence | 0.00 | 0.00 | 0.00 | 0.00 | 0.00 | 0.00 |
|  | Balanced Accuracy | 0.50 | 0.50 | 0.50 | 0.50 | 0.50 | 0.50 |

SI4: Confusion matrices of predicted behaviors from accelerometer data collected from California condors using 6 different supervised classification algorithms. Data are number of segments. Columns represent actual categories, rows represent predicted categories. Predictions were made without tuning of the models and using default model parameters. Model types are random forest (RF), k-nearest neighbor (kNN), neural network (NN), support vector machine (SVM), classification and regression tree (CART), and linear discriminant analysis (LDA).

Only the random forest and k-nearest neighbor models were subsequently tuned (see text for details).

| Model type |  |  | Reference | | | |
| --- | --- | --- | --- | --- | --- | --- |
|  | Prediction |  | Drinking | Flying | Sitting | Walking |
| RF | Drink |  | 0 | 0 | 0 | 0 |
|  | Fly |  | 690 | 26 | 10,903 | 232 |
|  | Sit |  | 290 | 12 | 33,714 | 149 |
|  | Walk |  | 0 | 0 | 0 | 0 |
|  |  |  |  |  |  |  |
| kNN | Drink |  | 16 | 0 | 41 | 1 |
|  | Fly |  | 740 | 28 | 13,093 | 178 |
|  | Sit |  | 224 | 10 | 31,483 | 202 |
|  | Walk |  | 0 | 0 | 0 | 0 |
|  |  |  |  |  |  |  |
| NN | Drink |  | 0 | 0 | 0 | 0 |
|  | Fly |  | 878 | 32 | 13,480 | 221 |
|  | Sit |  | 102 | 6 | 31,137 | 160 |
|  | Walk |  | 0 | 0 | 0 | 0 |
|  |  |  |  |  |  |  |
| SVM | Drink |  | 0 | 0 | 0 | 0 |
|  | Fly |  | 790 | 22 | 9,302 | 152 |
|  | Sit |  | 190 | 16 | 35,315 | 229 |
|  | Walk |  | 0 | 0 | 0 | 0 |
|  |  |  |  |  |  |  |
| CART | Drink |  | 0 | 0 | 0 | 0 |
|  | Fly |  | 610 | 24 | 11,019 | 225 |
|  | Sit |  | 370 | 14 | 33,598 | 156 |
|  | Walk |  | 0 | 0 | 0 | 0 |
|  |  |  |  |  |  |  |
| LDA | Drink |  | 0 | 0 | 0 | 0 |
|  | Fly |  | 250 | 16 | 10,659 | 156 |
|  | Sit |  | 730 | 22 | 33,949 | 225 |
|  | Walk |  | 0 | 0 | 9 | 0 |

SI5: Plot showing the optimal number of clusters (k=4) for K-mean clustering. See text for details on how we determined and visualized the optimal number of clusters using within cluster sums of squares.


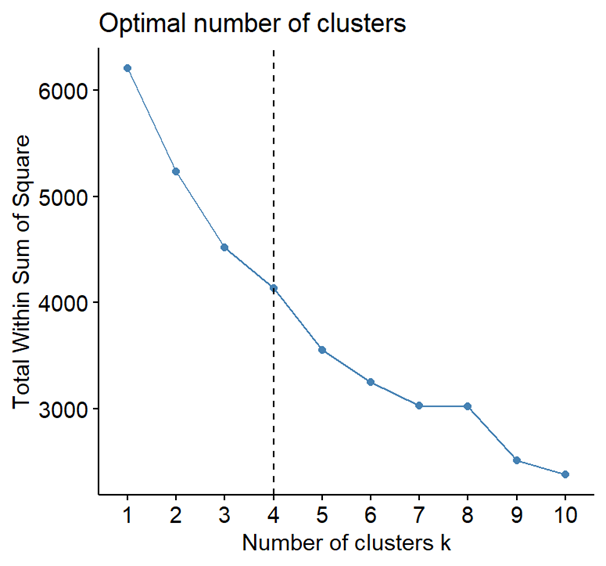

Supplement: Supplementary file 1 — Data S1: [file ECE3-13-e10035-s001.docx]
